# Caesarean delivery on maternal request: the perspective of the postpartum women

**DOI:** 10.1186/s12884-024-06464-5

**Published:** 2024-04-09

**Authors:** Célia J. L. Sitoe Muhandule, Cristine M. S. Benetti, Laura B. Fogulin, Silvana F. Bento, Eliana Amaral

**Affiliations:** 1 School of Medical Sciences, University of Campinas (UNICAMP), P.O. Box 6081, Campinas, SP 13084624 Brazil; 2 José A Pinotti Women’s Hospital, Center of Integral Services for the Health of Women (CAISM), University of Campinas (UNICAMP), Campinas, SP Brazil

**Keywords:** C-section on maternal request, Caesarean delivery on maternal request (CDMR), Elective C-section, Mode of delivery preference, Women´s choice childbirth

## Abstract

**Background:**

Caesarean delivery on maternal request (CDMR) is an increasing delivery option among women. As such, we aimed to understand the reasons that led pregnant women to request a caesarean delivery.

**Methods:**

A phenomenological study was conducted with semi-structured interviews, in a convenience sample, for women who had undergone a CDMR between March and June 2023, in a public reference university hospital in Campinas, Brazil. The interviews were recorded, transcribed and subjected to thematic analysis, supported by Nvivo®, and Reshape®.

**Results:**

We interviewed eighteen women between 21 and 43 years of age. The reasons for C-section as their choice were: 1) fear of labour pain, 2) fear for safety due to maternal or fetal risks, 3) traumatic previous birth experiences of the patient, family or friends 4) sense of control, and 5) lack of knowledge about the risks and benefits of C-section.

**Conclusions:**

The perception of C-section as the painless and safest way to give birth, the movement of giving voice and respecting the autonomy of pregnant women, as well as the national regulation, contribute to the increased rates of surgical abdominal delivery under request. Cultural change concerning childbirth and better counseling could support a more adequate informed decision-making about delivery mode.

## Background

Cesarean delivery on maternal request (CDMR), defined by the American College of Obstetricians and Gynecologists (ACOG) as a primary cesarean section at maternal request, in the absence of any maternal or fetal indication, performed after 39 completed weeks of gestation or with verification of pulmonary maturity [1], is a trend, following a movement to give women a voice and guarantee their right to choose [2, 3]. Despite the proven effectiveness of caesarean section in saving the life of the mother and/or fetus, in conditions where vaginal delivery is unsafe, performing a caesarean section exposes the pregnant woman and the fetus to health risks when it is unnecessary [1].

Worldwide, CDMR is responsible for an increasing proportion of caesarean sections, estimated at 0.2% to 42% of all caesarean sections, varying according to the sociodemographic characteristics of the women and the care profile of the maternity hospital where the birth takes place [4]. In Brazil, where the caesarean section rate is 56%, ranging from 40% in the public sector to 84% in the private sector, the proportion of CDMR is not known [2]. Studies suggest that the CDMR contributes to an increase in the caesarean section rate in general and in Brazil, where there is a culture of caesarean sections, this tendency is stronger, in addition to the cases of those who, in the absence of a maternal or fetal indication, take advantage of the caesarean section to carry out surgical sterilization. In Brazil, this type of surgery used to be restricted to the moment of childbirth, but has recently been legalized during delivery [5–7].

CDMR brings ethical tensions for service providers, since it is defined as a potentially unnecessary caesarean Sect. [8] and with evidence of increased maternal risk [9]. For women, elective caesarean section is associated with the risk of hysterectomy, mortality, hospitalization, abnormal placentation and uterine rupture [10]. There are additional risks for the newborn and the child, such as admission to the neonatal intensive care unit, infections, persistent verbal delay, infant mortality, neonatal death, asthma and obesity [10–12]. On the other hand, CDMR is associated with a lower chance of urinary incontinence in the first year postpartum and fetal brachial plexus injuries [1, 13]. Some studies consider obstetricians' acceptance of this type of delivery to be a defensive clinical practice [14].

CDMR is more prevalent among women with certain demographic characteristics, such as a higher level of education, economic power, access to health insurance, advanced maternal age, living in areas with a notable level of socio-economic development [4, 15]. Thus, the option of caesarean section could be more accessible to a certain group of women, which would reinforce health inequalities [11], given the World Health Organization's concern that caesarean sections should be performed when necessary for clinical reasons or out of maternal conviction, after extensive counseling based on the best evidence, without aiming for certain caesarean section rates [16].

It is therefore pertinent to explore women's perspectives on CDMR in order to understand the reasons for requesting a caesarean section and potential support strategies for preventing avoidable caesarean sections. This study aims to understand the reasons for CDMR among women giving birth in a public academic maternity hospital in Brazil, where legislation allows and facilitates access to this option for all women.

## Methods

### Study design

This is a qualitative study with a phenomenological approach, using in-depth interviews. Convenience sampling was used. The data was analyzed according to the psychological and human science method proposed by Amadeo Giorgi, based on Edmund Husserl's phenomenology [9, 10]. Phenomenology aims to describe the essence of the meanings expressed by the participants about their experiences, distancing itself from pre-established theories, beliefs or criticisms. The researcher puts the world in brackets, stripping away the necessary judgments about what is happening and how the other person expresses their experience in order to understand the peculiar and unique way of the reported experience, also called phenomenological reduction [17, 18].

### Participants

The study was carried out with puerperal women who underwent CDMR between March and June 2023, at a tertiary and quaternary referral university hospital, which provides medium and high complexity care for women and newborns through the public health system, It performs an average of 1,000 deliveries a year, with an overall caesarean section rate of 59.7%, and its catchment area covers 42 municipalities in the state of São Paulo and almost five million people, in addition to demands from other regions.

The inclusion criteria were: being a puerperal woman undergoing a caesarean section at the mother's request, being at least eighteen years old and agreeing to record the interview. The exclusion criteria were: being a woman undergoing caesarean section at the mother's request, under the age of eighteen or not agreeing to record the interview.

To recruit participants, the principal investigator (CSM) accessed the electronic database system of the referral hospital. After identifying all the women who had had a cesarean section the previous day, she reviewed the indications for abdominal delivery. All women who had registered CDMR were invited to participate. The Informed Consent Form was presented, the research project was presented and all doubts were clarified. Those who agreed to take part signed the Free and Informed Consent Form before the interviews.

To carry out the study, the ethical standards for research involving human beings in Brazil were followed and the research project was approved by the Research Ethics Committee of the State University of Campinas (UNICAMP), under CAAE: 65052722000005404.

### Data collection

The interviews were carried out by the researcher (CSM), a Mozambican national who has a degree in public health and is inserted in a different cultural context to Brazil, as far as caesarean sections are concerned. The interviews took place in a private room in the postpartum ward. The women had the option of bringing their babies into the interview room or leaving them with their companions if they felt safe.

An interview script consisting of open-ended guiding questions about previous childbirth experience and the reasons for requesting a caesarean section during the current pregnancy was developed and discussed by the researchers. The interviews were carried out until the information became saturated [19]. The women's recorded responses were later transcribed using Reshape, an online platform that supports audio transcription. The interview conducted two days after the birth lasted an average of 22 min (11 to 32 min).

### Data analysis

Thematic analysis followed the method proposed by Braun and Clark [20], consisting of a six-step structure for identifying and analyzing patterns. It was supported by Nvivo12® software.

**Fig. 1:**
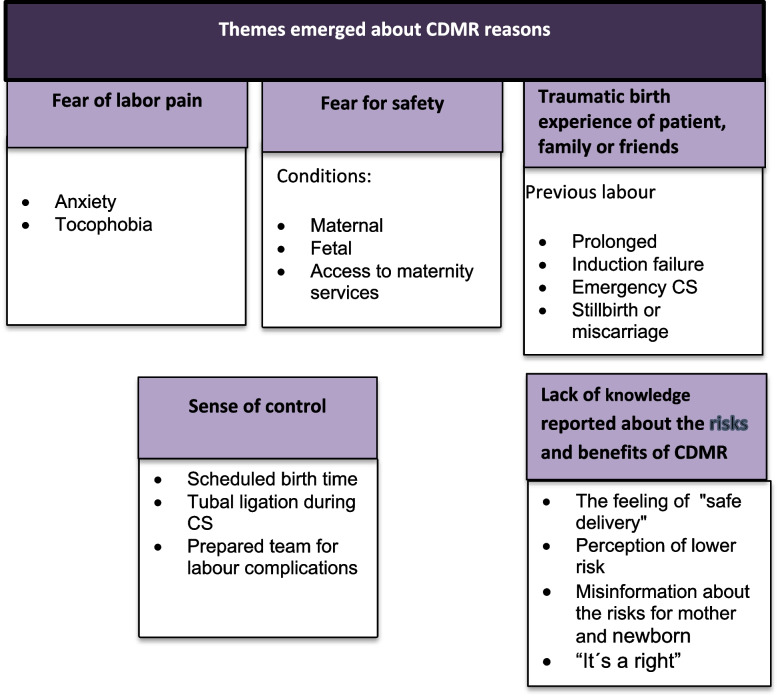
Themes emerged from reasons for women's choice on CDMR

The researcher (CSM) carried out the six recommended steps: 1. Familiarization with the data (the transcripts were read thoroughly to get a general impression); 2. Generation of initial codes (single units of themes were identified in the transcribed text, consisting of one or more sentences or paragraphs); 3. Search for themes (the units of meaning were reflected on and thematized according to the women's point of view); 4. The thematized units of meaning were reviewed and condensed; 5. Definition and naming of the themes (themes that emerged)—Fig. 1; 6. The themes that emerged in relation to the phenomenon studied were described.

The variables contained in Tables 1 and 2 referring to the sociodemographic and reproductive data obtained from the women were defined as follows:

- Age: The woman's age, self-reported in the interview, in whole numbers.
- Skin color/ethnicity: the woman's skin color, self-reported in the interview, categorized as white and non-white (non-white includes women who self-report brown, black, yellow and other).
- Marital status: indicates current marital status. Categorized as: single, married, in a stable union, divorced and widowed.
- Family income: number of minimum wages of the individual income of the residents in the woman's household, self-reported in the interview, expressed in dollars.
- Schooling: assessed by the last grade completed at school, reported in the interview; categorized in years completed.
- City of origin: whether or not the patient lives in the same city as the maternity hospital. Whether or not the patient lives in the same city as the institution where she is hospitalized.
- Parity: refers to the number of births or abortions prior to the birth under study. We categorized primiparous women as those who had never given birth and multiparous women as those with a history of any number of previous births or abortions.
- Gestational age at the time of the caesarean section: gestational age, identified by the difference in weeks from the date of the last menstrual period and/or obstetric ultrasound ideally carried out up to the second trimester, recorded in the woman's medical records on the date of delivery. Variable obtained from medical records, described in completed weeks, categorized as 39 completed weeks (yes or no). Previous caesarean section: number of caesarean sections carried out before the birth under study, categorized as yes (for one or more) and no (for none) according to the record in the medical chart.

**Table 1:** Sociodemographic characteristics of the women

Variables	n
**Age**	**18**
21–30 years	9
31–40 years	7
+ 41 years	2
**Schooling**	**18**
1–5 years	1
5–9 years	14
+ 9 years	3
**Marital status**	18
Married	11
Single	4
Co-habiting	3
**Family income**	18
One minimum wage (250 US$)	10
+ one minimum wage (250 US$)	8
**Skin color**	18
White	11
Non-white	7
**Works outside the home**	18
Yes	13
No	5
**Residence Same city as maternity hospital**	18
Yes	3
No	15

**Table 2:** Women's reproductive characteristics

Variables	N
**Parity**	18
Primiparous	6
Multiparous	12
**Previous cesarean section**	12
**Outcome of previous pregnancy**	12
Miscarriage	5
Neonatal death	1
Born-alive	6
**Gestational age of current pregnancy (39 completed weeks)**	18
Yes	8
No	10
**Morbidity in current pregnancy**	18
Yes	17
No	1
**Tubal ligation during current delivery**	18
Yes	5
No	13

## Results

Of the 28 women eligible for inclusion, six refused to take part and four were excluded for not agreeing to record the interview. In total, the information collected from the interviews with 18 women aged between 21 and 43 was analyzed. All of them had some clinical or obstetric complication, the main ones being diabetes and hypertension. Only one of the women did not have any morbidity, despite the hospital being a referral center for high-risk cases. This was because she arrived on demand for urgent care. Fourteen women had at least nine years of schooling, fourteen had a steady partner (married and cohabiting) and only three lived in the city where the hospital is located. Two-thirds (twelve) of the women interviewed had a previous caesarean section, a current caesarean section was carried out before 39 weeks in ten of the 18 women and a few (five) had a tubal ligation during their current delivery.

The main sociodemographic and reproductive characteristics of these women are shown in Tables 1 and 2.

The reasons for CDMR reported by the postpartum women are presented in the following themes:

### Fear of labor pain

Many women opted for a caesarean section because they were afraid of the pain of childbirth. This fear arose from unpleasant experiences told to them by family members and people close to them. These reports made them think that they would be unable to tolerate the pain of childbirth.

"I was afraid of the pain of childbirth, I heard that it hurt a lot, people said I couldn't take it, everyone said that it stayed in my head, you know." (P7, first CS).

Multiparous women also reported fear of pain as one of the reasons for the CDMR, despite having gone through labor, they still feared the pain of childbirth. In addition, the previous caesarean section was remembered as a peaceful birth.

"My caesarean is because of fear. Because normal is… They say the recovery is quicker afterwards, but it's a lot of suffering. Many hours, some women suffer for hours. I didn't want to know… the experiences I've heard and lived through weren't so good, I think that's what I was left with. So I didn't want to know. Very few people say that natural childbirth is good. Oh, it came out so quickly I didn't even notice. It's one in a million. " (P21, second CS).

### Feeling of safety due to maternal or fetal risks

Other women preferred CDMR because they felt it was the safest option, especially when they realized that there was an anomaly in them or in the fetus. The majority had morbidity (ies), whether or not they were due to the pregnancy, and feared that they wouldn't have time to get to the referral maternity hospital if they went into spontaneous labor, as they lived far away.

"…I wanted a caesarean section because I had a transplant and I'm scared too, right? Scared. As I live far away, I was afraid I wouldn't make it. Going into labor early, not being able to get here, right? Because it was half an hour away and something was going to happen. So I wanted to plan the right day for me to come so that there would be no risk." (P6, First CS).

### Traumatic birth experience of the patient, family or friends

Nulliparous women, with whom family and friends shared negative birth experiences, had the idea that vaginal birth brought a lot of suffering.

"Yes, my mother actually encouraged me. She has two children from normal pregnancies and she said it was difficult, sometimes she might have to pull her child, breaking some bones. She said to have a cesarean section because it's better. I even have a friend of mine who tried to induce and she went to the shower, went to the ball and couldn't do it. There was no dilation, right? Then, finally, in the last case, they did a caesarean section. Listening to her scared me too." (P14 first CS).

Multiparous women who had negative experiences in their previous delivery, such as slow progression of labor, accompanied by painful contractions without dilation and failure to induce labor, no longer wanted to try vaginal delivery.

"Because I kind of already knew what was going to happen… I didn't want to go through everything, right? The pain, the suffering, right? But recovery is also difficult, because my body is hard to heal. Now I'm conscious, I was able to enjoy the birth… I wanted to enjoy this moment." (P3 second CS).

### Feeling of control

For some women, caesarean section is a way of giving birth that has proved to be more practical and in line with their life dynamics. This mode of delivery allowed for better planning and the possibility of scheduling the day of birth, as well as considering it quick and minimizing the feeling of unpredictability. Some women decided to undergo CDMR in order to undergo tubal ligation at the same time.

"… I don't have time, I'm quite honest, my time is very, very limited… I want to have a cesarean section, ok……You've made an appointment, it's on the right date, you go and do it. No vaginal delivery, you have to wait for the urge to happen." (P4, first CS).

### Lack of knowledge about the risks and benefits of cesarean delivery

Reports from some women show that they do not have clear knowledge about the risks and benefits of cesarean delivery. Many consider doctors' advice on the method of delivery to be a way of scaring them. Even those who were able to recall the information they had received had their choice of caesarean section clearly defined.

"Because they knew about the C-section in a terrible way. They talked, talked, talked, oh, that, you can take the risk of that. They said it could happen, right? Then in the end you commit yourself, you sign the paper, like, you're going to die…" (P 21 s CS).

## Discussion

The main reasons given by women for having a cesarean section were fear of the pain of childbirth, which they felt they would not be able to bear, based on reports of unpleasant experiences heard from family and friends. Another reason was the belief that a caesarean section would be safer in cases where there was evidence or suspicion that the baby had an anomaly. As many of the women lived in other municipalities, they were afraid that when the signs of labor began, they wouldn't be able to get to the hospital in time because of the distance. Choosing a caesarean section would be a way of protecting the baby from any risks that could harm it. Mention was also made of women's lack of knowledge about the risks and benefits of caesarean section. All the women interviewed, except one, had morbidities, and these clinical situations generated a lot of fear and anxiety, even though they were controlled morbidities that did not contraindicate vaginal delivery. The pregnant women didn't want to suffer and/or lose control of the situation in terms of the best possible care for themselves and their babies. Although our findings reflect the practices of a specific population, they are in line with the international debate on the subject [21].

In Brazil, since 2019, cesarean section legislation has guaranteed pregnant women the right, in elective situations, to opt for a cesarean section after 39 completed weeks of pregnancy, similar to the ACOG recommendation [1]. However, in our sample, many had a CDMR even before 39 weeks, a situation that goes beyond the strict definitions in the literature and regulations mentioned above.

On the other hand, the risks of caesarean section versus vaginal delivery seemed to be insufficiently discussed or reflected on by these women, as the results showed, preventing a truly informed decision. In view of this finding, it is necessary for health services to offer more information to pregnant women during prenatal care about the types of births and the risks and benefits involved for both mother and baby, as a strategy for reducing unnecessary caesarean Sects [22, 23].

The long-standing and well-established culture of caesarean sections in Brazil, which is passed down from generation to generation, generates a sense of security for the mother and child, of a pain-free birth, of comfort because it is programmable and an opportunity to take advantage of the moment of the caesarean section to have a tubal ligation. On the other hand, doctors also prefer this type of delivery due to the heavy workload they have and in this case it is possible to schedule the day and time of the baby's birth, without the baby being available to the woman for too long in hospital. What's more, cesarean sections are perceived by professionals as having fewer risks and consequently fewer lawsuits. Faced with this scenario, caesarean sections are normalized as a natural form of childbirth, especially among women from higher socioeconomic groups, women with more years of schooling and white women [14]. This profile fits in with some of the characteristics of the participants in this study.

Another point to raise is that obstetric violence is a topic that is increasingly being debated, including in Brazil. This debate has led women and health professionals to reflect on their choices, rights, responsibilities and desires, since the fear of vaginal childbirth is also due to lived or reported experiences that mirror obstetric violence [24] in general and associated with limited access to analgesia to minimize the pain of vaginal childbirth.

A limitation of this study is the fact that the interviews were conducted with women treated at a tertiary referral service, generating a sample of women with clinical and gestational complications, many of whom had had a previous caesarean section. Therefore, the results of this study offer some light, but cannot be extrapolated to the general population of pregnant women. "the results offer some light, but are exploratory as this is a qualitative study".

## Conclusion

In this context, continuing education for women on the risks and benefits of both types of delivery should be promoted, as a way of bringing more knowledge that can promote a change in the culture of the Brazilian population. This same educational work should be carried out with doctors so that the best decision can always be made about the type of delivery in the light of each woman's clinical condition, since each one has a different clinical history. Always focusing on the care of the pregnant woman and her baby. This is a process of changing paradigms that could, in the long term, change the way people think and, consequently, the Brazilian culture on this issue.

## Data Availability

The datasets generated and/or analyzed during the current study are not publicly available due to ensuring confidentiality of participant data, but are available from the corresponding author upon reasonable request.

## Abbreviations

UNICAMP: University State of Campinas
CAISM: Prof. Dr. José A Pinotti Women’s Hospital, Center of Integral Assistance for Women´s Health
CDMR: Caesarean delivery on maternal request
ACOG: American College of Obstetricians and Gynecologists
CS: Cesarean section
